# Hypertension and its Correlates in the Oldest Old Population Aged 80 Years and Above in Urban South India

**DOI:** 10.4172/2167-7182.1000472

**Published:** 2018-05-31

**Authors:** Bana Manishaa Reddy, Enakshi Ganguly, Pawan Kumar Sharma

**Affiliations:** Department of Community Medicine, MediCiti Institute of Medical Sciences, MIMS Campus, Ghanpur, Ranga Reddy District, Telangana, India

**Keywords:** Oldest old, Hypertension, Correlates, Prevalence, Uncontrolled hypertension, Community

## Abstract

**Background:**

Hypertension is a major problem among the geriatric population, presenting the risk of multiple associated co-morbidities and organ system damage. Data related to the epidemiology of controlled and uncontrolled hypertension among the oldest old population is sparse, more so from developing countries. The objectives of the present paper were to identify the prevalence and correlates of hypertension according to gender among the urban community-dwelling healthy oldest old population.

**Methods:**

200 healthy people aged 80 years and above were recruited by random selection from Hyderabad city of South India in 2017. A trained investigator collected data on background history, history of hypertension and other co-morbidities, medications and sleep. Participants were tested for muscle strength, gait speed, and SPPB and cognitive impairment.

**Results:**

The prevalence of hypertension was 83.5%; 81.6% among men and 84.7% among women. 64.5% was self-reported. Hypertension was controlled with treatment among only 46.2%. In 74.6%, it was controlled even without treatment. The independent correlates were BMI per SD increase (OR: 1.92, 95% CI: 1.17–3.16), diabetes (OR: 6.02, 95% CI: 1.24–29.11) and asthma (OR: 3.59, 95% CI: 1.05–12.29). Among men, BMI per SD increase was significantly associated while hemoglobin per SD increase, height per SD decrease, and arthritis were positively associated among women. Increasing heart rate among total subjects (OR: 0.44, 95% CI: 0.27–0.71), and among women (OR: 0.47, 95% CI: 0.24–0.92) showed a negative association.

**Conclusion:**

The prevalence of hypertension was high. The correlates were different for men and women. Subjects were unaware of their control status which posed an increased risk for organ damage, and development of co-morbidities. Policies aimed at improving quality of life of the oldest old should place due stress on appropriate hypertension management in developing countries.

## Introduction

Hypertension is one of the most common morbidity in the older age groups significantly impacting their health conditions [1]. According to the Seventh Report of the Joint National Committee on Prevention, Detection, Evaluation and Treatment of High Blood Pressure (JNC-7), hypertension affects more than two-thirds of individuals aged 65 years and above [2]. Globally, 7.1 million deaths per year are attributable to hypertension among the estimated 1 billion individuals suffering from the condition [3]. Suboptimal Blood Pressure (BP) (>115 mmHg) has been considered to be the most important attributable risk factor for deaths worldwide [4]. Despite the gravity of the consequences, the awareness of the population about this silent killer is unsatisfactory. In the US, the awareness of hypertension is only 70%, treatment received is 59%, and controlled hypertension is only 34% [2]. Most of the studies on hypertension have been done on adults between >18 years to 59 years of age, and therefore are inadequate to describe the epidemiology of hypertension among older hypertensive patients who are highly heterogeneous, possessing widely variable physiological ability and vulnerability even for individuals of the same age [5].

Oldest old (>80) are expected to increase to 434 million by 2050 [6]. With the progression of the aging population, diseases of the elderly have become the center of attention in most developed countries, with many reporting the rising prevalence of chronic disorders amongst them. Data from the Framingham Heart Study, in men and women free of hypertension at 55 years of age indicated that the remaining lifetime risks for development of hypertension through 80 years were 93% and 91% respectively [7]. The severity of hypertension increases markedly with advancing age in women as well. Studies highlighting the age and gender differentials in the progression of hypertension showed its prevalence to be less in women than in men until 45 years of age, then similar in both sexes from 45 to 64 and subsequently much higher in women than men over the age of 65 years [8]. Research supports the fact that most geriatric women (age 60–79 years: 48.8%; age ≥ 80 years: 63%) have stage 2 hypertension (BP ≥ 160/100 mmHg) or receive antihypertensive therapy [9–11].

Hypertension is the main risk factor for most of the morbidities in older age including cardiovascular and cerebrovascular diseases, poor quality of life and poor activities of daily living (ADL) [12]. Numerous studies have demonstrated risk for stroke, left ventricular hypertrophy, congestive heart failure, coronary and peripheral artery diseases, vision impairment, end-stage renal disease, cognitive impairment, and dementia among hypertensives [13,14]. Additionally, hypertension has adverse effects on most organ systems including cerebrovascular, cardiovascular, renal, ocular and vascular etc. [13,15,16]. Although both systolic blood pressure (SBP) and diastolic blood pressure (DBP) are established risk factors, with advancing age, SBP becomes a better predictor than DBP, of cardiovascular disease and other co-morbidities [17,18]. The intricacies in the presentation and outcome of hypertension, and its associations with diverse risk factors among elderly, make it a complicated disease warranting optimal control and persistent adherence to prescribed medication to reduce the risks of cardiovascular, cerebrovascular and renal disease [1].

While the literature on hypertension prevalence and its predictors among the geriatric population abound from the Western countries, we could not find comparable literature from India where the burden of chronic diseases, including hypertension, among the geriatric population is presumed to be growing at a fast pace. We also hypothesize that the dynamics of hypertension in oldest old Indians is different than the contemporary western population. We, therefore, planned to study the prevalence of hypertension and its correlates in the oldest old population of urban Hyderabad with the objectives to determine the prevalence of hypertension, identify its correlates and study the gender differences in prevalence and correlates of hypertension in healthy oldest old population aged 80 years and above residing in urban community of a South Indian state.

## Materials and Methods

We analyzed the data from a cross-sectional study done primarily on frailty among the oldest old population, aged 80 years and above, community-dwelling apparently healthy persons from south-central part of India, Hyderabad city, Telangana state, in 2017. We enrolled 200 subjects, 76 (38%) men and 124 (62%) women who consented to participate.

The individuals were randomly enrolled from 12 townships (residential gated communities, population ranging from 1000 – 6000) located in different geographic locations of Hyderabad city. The data was collected by house-to-house visits by the trained investigator. A list of households was prepared with the help of societies of the townships having at least one age-eligible subject in the household. A random number was assigned generated by random number generator software to each household. All eligible subjects agreeing to participate, from the selected household were included in the study. The study was ethically approved by Institutional Review Board (IRB) of Mediciti Institute of Medical Sciences, Ghanpur, Hyderabad and Indian Council of Medical Research (ICMR), Delhi. All the subjects were required to give written informed consent, translated in the local language, and explained by the investigator. Inclusion criteria were Indian nationality, age ≥ 80 years, living in an urban area of Hyderabad city, possessing cognitive ability to understand the investigator’s instructions, and consenting to participate. Those suffering from known debilitating chronic disease, mental illness, or terminal illness were excluded.

### Measurement of blood pressure

Upon visit, resting blood pressure was measured thrice with a 1minute interval using an electronic sphygmomanometer (OMRON HEM 7120, Omron Healthcare Co., Ltd., Japan). Systolic and diastolic blood pressures were recorded on relaxed calm participants in the sitting position with their elbows raised at the level of their heart. They were instructed to abstain from eating, drinking alcohol or caffeinated drinks or exercise at least for 30 min before blood pressure measurement. The average of last two readings was used to define systolic and diastolic blood pressures [1].

### Definitions

Hypertension was defined as having an SBP ≥ 140 mmHg, DBP≥ 90 mmHg, and/or self-reported and /or taking anti-hypertensive medicine, prior diagnosed by the doctor and/or taking anti-hypertensives [2]. Treatment of hypertension was defined as the current pharmacological treatment initiated by the doctors for lowering elevated BP.

Controlled hypertension was defined as having a current SBP <140 mmHg and DBP <90 mmHg associated with the pharmacological treatment or diagnosed by the doctor and having a current SBP <140 mmHg and DBP <90 mmHg; and uncontrolled hypertension was defined as SBP ≥ 140 mmHg, and DBP ≥ 90 mmHg and taking pharmacological treatments OR diagnosed hypertensive by the doctor, not taking pharmacological treatment and SBP ≥ 140 mmHg, and DBP ≥ 90 mmHg. Undiagnosed hypertension was defined as SBP ≥ 140 mmHg, a DBP ≥ 90 mmHg but never diagnosed with hypertension previously by the doctor nor taking pharmacological treatments.

We collected information on age, sex, marital status, education, self-reported general health, medical history, physical function and chronic diseases and disabilities by using a questionnaire developed by adapting questions from leading international studies WHO Study of Global AGEing and Adult Health (SAGE) [19], Mobility and Independent Living in Elders Study (MILES) [20] and the Lifestyle Interventions and Independence for Elders (LIFE) pilot study [21] questionnaires. The depression was assessed by 15 item Geriatric Depression Scale (GDS) [22]. Cognitive function was assessed by Mini-Mental Scale Examination scale (MMSE) [23]. Activities of daily living constituted of 7 activities that included walking across the small room, bathing, grooming, dressing, eating, getting out of bed and into a chair and toilet [24]. All co-morbidities were self-reported.

The short physical performance battery (SPPB) consisting of a 4m walk, repeated chair stands, and three hierarchical standing balance tests were performed. Each of the three performance measures was assigned a categorical score ranging from 0 to 4, with 4 indicating the highest level of performance and 0 the inability to complete the test. A summary score ranging from 0 (worst performers) to 12 (best performers) was calculated by adding gait speed, chair stands, and balance scores [20]. Hand grip strength was measured by Jamar dynamometer using standard protocol [20]. An average of the last two readings out of three on the dominant hand was considered as the participant’s grip strength.

Data were analyzed using SPSS 21.0 (SPSS Inc., Chicago, IL, USA). We reported characteristics and correlates of hypertensive and non-hypertensive subjects as mean, standard deviation and proportions. We compared categorical variables by using chi-square test and continuous variables by t-test. The variables that emerged statistically significant (p<0.10) in univariate analysis were considered for multivariate models. For this communication, data and calculated effect sizes were used to perform post-hoc power analyses on prevalence of hypertension; an a priori power analysis was not possible for the lack of pilot data. Post-hoc power analysis was performed using SPSS 21.0.

We created three models for logistic regression: men, women and total population adjusting for sex. We performed backward elimination logistic regression testing, the deletion of each variable using a chosen model comparison criterion, deleting the variable that improved the model the most and repeating this process until no further improvement was possible, to get final set of the independent predictor variables. The variables having p<0.05 (considered significant) were reported as independent predictor variables. The results of logistic regression were presented as odds ratios (OR) with 95% confidence interval (95% CI).

## Results

The baseline characteristics of the study population are shown in Table 1. There were 200 subjects (38% men and 62% women), having a mean age of 83.44 ± 3.87 years (Table 1). Total hypertensive individuals were 83.5%. 81.6% of men and 84.7% of all women had hypertension.

Hypertension was controlled with treatment among 46.2% individuals amongst the 80% having diagnosed hypertension. For 53.8%, hypertension was uncontrolled even with treatment (Table 2). Table 2 shows the blood pressure distribution of the subjects.

Upon univariate analysis, on anthropometry, mean BMI was significantly higher among men, women and total participants with hypertension compared to those without (Table 3). Women without hypertension were significantly shorter and heavier than their hypertensive counterparts. Women with hypertension, as also total, had significantly higher waist and hip circumferences compared with normotensive women. Hypertensive men had higher weight and hip circumference than non-hypertensive men. The gender differences between men and women having hypertension were significant for height and hip circumference.

Men with hypertension had significantly higher proportions of diabetes, obesity, low energy and poor sleep compared with those whose BP was not high. Hypertensive women, on the other hand, had multiple comorbidities in significant proportions that included asthma, arthritis, and stroke in comparison to normotensive women, who had significantly more anemia.

Upon logistic regression, BMI per 1 SD increase was associated 2 folds (OR: 1.92, 95% CI: 1.17–3.16), diabetes 6 folds (OR: 6.02, 95% CI: 1.24–29.11) and asthma 3 folds (OR: 3.59, 95% CI: 1.05–12.29) with hypertension (Table 4). BMI per 1SD increase among men (Table 5) and hemoglobin per 1 SD increase, height per 1SD decrease, arthritis among women (Table 6) were strongly positively associated with hypertension. Increasing heart rate among total subjects (OR: 0.44, 95% CI: 0.27–0.71), as also among women (OR: 0.47, 95% CI: 0.24–0.92) appeared protective against hypertension.

## Discussion

### Prevalence of hypertension

The prevalence of hypertension among older population aged 80 years and more in our study was 83.5%. About 65% of hypertension was self-reported. Our prevalence was higher when compared with United States in Framingham Heart study (74%) [10] and NHANES data (76.5% ) [25], Korea (71%) [26], China (36.2%) [27] and Spain (72.8%) [28]; and lower compared to France (97.2%) [29] and thirteen HYVET study countries (89.9%) [12]. One study in rural India reported a prevalence of 61% in a subset analysis, which was lower than ours [30].

Isolated systolic hypertension (ISH) was found to be 27.5% in the present study, using the JNC VII definition. When compared with the Framingham Heart study ISH rates of 18% (80–89 years) and 25% ( > 90 years) [31], our rates are higher. This difference is primarily due to the higher cut off values (SBP>160 and DBP >95 mm Hg) used by the Framingham study. Similarly, the Systolic Hypertension in the Elderly Program (SHEP) study also used higher cut offs, defining ISH as an SBP of >160 mm Hg with a DBP of <90 mm Hg, and reported the prevalence as 22% among 80-year-olds [32].

Systolic diastolic hypertension, detected in 22% population in our study, was considerably lower than US population where a prevalence rate of 76.5% was reported between years 2005-10 [25].

### Controlled and uncontrolled hypertension

About 54% of the hypertensives receiving antihypertensive treatment in the present study were found to have uncontrolled hypertension. Additionally, about 25% had uncontrolled hypertension and were not on treatment. Our prevalence of uncontrolled hypertension was higher than 37% prevalence reported by a French study [29]. NHANES data also showed about 55% prevalence of uncontrolled hypertension with treatment among >85-year-olds [33]. Hypertension was diagnosed in about 80% of the population. The awareness rate of 93% reported from Spain was much higher compared with ours [28] 25% of the elderly in the present inquiry who had uncontrolled hypertension did not receive any treatment.

### Comorbidities

The SAGE study reported the following comorbidities among Chinese aged 80 years old and above: arthritis 25.5%, stroke 7.1%, angina 14.5%, diabetes 7.7%, chronic lung disease 13.2%, asthma 3%, depression 0.1%, and hypertension 37.3% [34]. These rates were much lower compared to our population where arthritis was 45%, stroke 60%, diabetes 24%, asthma 26%, and depression 49% among hypertensives. Multimorbidity among hypertensives aged 80 years and older is of particular concern because it presents the risk of overtreatment and polypharmacy that has been shown to increase the mortality rates among them. The Jerusalem longitudinal study showed no difference in mortality between normotensive, untreated and treated hypertensive subjects according to sex. Their analysis of 85-year-old hypertensives showed that subjects with controlled SBP tended to have a worse survival, before adjustment for comorbidities [35]. Though we did not study the survival of the hypertensives in the present study, we expect similar 5-year mortality due to complications of comorbidities affecting the effectiveness of hypertension treatment, as reported in HYVET where the benefits of antihypertensive medication for risk reduction of stroke were countered by increased all-cause mortality [12].

### Correlates of hypertension

BMI increase per 1 SD was independently associated with hypertension in our population, and among men. BMI has been shown to be significantly associated with systolic and diastolic blood pressure among 80 years and older Japanese after controlling for factors known to influence blood pressure values, such as sex, alcohol intake, current smoking status and serum glucose, total cholesterol and creatinine concentrations [36]. The BELFAST study also reported BMI per 1SD to have a significant association (OR: 1.28) with hypertension [37]. The strongest association between BMI and hypertension among 80 years and older have so far been reported among the US population from NHANES data (OR: 42.6; 95% CI: 30.5–59.6) [38].

Increasing heart rate per 1 SD showed a negative association with hypertension among the total population and among women in our study. Resting heart rate (RHR) is gaining acceptance as an independent cardiovascular risk factor [39]. Among the few studies to study associations between heart rate and cardiovascular risks, Park et al. have shown a significant positive association between increasing resting heart rate quartiles and arterial stiffness among Korean adults (mean age 54 years) [40]. Age-adjusted RHR >66 was reported to be positively associated with an increase in DBP, but not SBP among 113 Brazilian men and women aged 80 years and above [41]. This discordance may be due to the difference in mean heart rate measured in their population and ours. Studies on healthy young populations have fairly established that increases in heart rate amount to development of future cardiovascular risks. Clinical trials aimed at lowering heart rate in an attempt to lower blood pressure have yielded inconsistent results with none of the drugs in current usage demonstrating clear benefits [42]. The limitation of these trials is non-inclusion of older age groups, hence it may be theorized that the true effect of heart rate on hypertension among ages 80+ years are largely unknown. Since our study showed a significant inverse association between mean heart rate and hypertension, increasing heart rate through intervention may show some benefit for hypertension control in our population. It may also be hypothesized that drop in blood pressures may ultimately lower the heart rate among this population, which remains amenable to testing through longitudinal research. Further studies may be required to explore the relationship of lower heart rate and hypertension in the oldest old.

Asthma showed a three-fold increase in the risk of hypertension in this population. Asthma has been shown to be positively associated with hypertension among all ages by Salako and Ajayi [43]. Battaglia et al. have stated that comorbidities and resultant polypharmacy tend to influence the metabolism and excretion of respiratory drugs, and can negatively impact adherence to and persistence with chronic treatment with respect to asthma among elderly, or geriatric asthma [44]. Further, few studies have also suggested a patho-genetic link between metabolic syndrome and asthma through the pro-inflammatory low-density lipoproteins [45,46].

Diabetes is commonly associated with hypertension among elderly, with most international bodies recommending non-aggressive control of systolic hypertension in octogenarians having coexisting diabetes. Senior et al. reported 11% prevalence of diabetes among hypertensive elderly aged 80 years and older from New Zealand [47]. They, however, did not find diabetes to be independently associated with hypertension control; nevertheless, nearly half of their hypertensive patients had evidence of target organ damage, which together with diabetes mellitus was positively associated with treatment status, leading to the assumption that controlling blood pressure was more difficult in patients aged 80 years and above with diabetes.

Among women, hemoglobin per 1SD increase was associated with hypertension in our study, but not among men. Atsma et al. have shown hemoglobin to be positively associated with high blood pressure among younger healthy men and women [48]. Zakai et al. however, found hemoglobin drop to be associated with hypertension in elderly [49]. Few others have reported independent associations of hemoglobin with hypertension and gender among other risk factors [50]. Higher hemoglobin levels have been shown to be associated with hypertension among patients with early CKD, albeit younger than ours. The presence of CKD in a large proportion of Indian elderly (about 40%) [51] may justify our finding.

The association of height and hypertension amongst elderly women may be explained by the relatively high prevalence of osteoporosis (more than 50%) among community-dwelling urban elder South Indian women [52]. Loss of height in osteoporosis has been previously described [53], thereby suggesting the underlying association between hypertension and osteoporosis, both of which are documented risk factors for cardiovascular morbidity and mortality amongst elderly [54,55].

Self-reported arthritis was an independent risk factor for hypertension in this study. As shown in other studies, arthritis is associated with hypertension since both diseases have common risk factors including aging [56]. Research also indicates metabolic syndrome and aging-related phenotypes for osteoarthritis, which explains the link between all three conditions in our study [57,58].

### Strengths and limitations

This is the first population-based study from India, to our knowledge, and amongst the very few globally, to report findings focussed on the oldest old geriatric population aged 80 years and more. The study was carefully designed and an adequate sample was included to state the findings with sufficient power. Post hoc analysis established that the present study was well-powered (90.4%) to detect differences in proportions between hypertensive and non-hypertensive population. However, the cross-sectional nature of the study limits causal association to be drawn with the identified correlates. Further, an element of recall bias was inevitable owing to the age of the participants. The investigator was trained to minimize such bias to the maximum possible limit by allowing sufficient time for recollecting and interviewing in privacy to reduce interference with memory.

## Conclusion

The prevalence of hypertension among elderly aged 80 years and above was high. Women had a significantly higher prevalence than men. The correlates were different in men and women. The relationship of hypertension and various comorbidities including depression, decreased activities of daily living, cognitive impairment, frailty, cardiovascular diseases among men and anemia among women seemed to have reversed in comparison with Western literature and less than 80-year-olds.

## Figures and Tables

**Table 1:** Basic characteristics of the study population.

Characteristics	Men (N=76)	Women (N=124)	Total (N=200)	P-value
Age (mean ± SD)	83.78 ± 4.04	83.24 ± 3.76	83.44 ± 3.87	0.34
BMI (mean ± SD)	22.31 ± 4.31	23.22 ± 4.75	22.88 ± 4.06	0.18
BMI (normal/ overweight/ obese) (%)	43.7	42.5	42.9	0.49
Marital status (currently married/unmarried/widowed) (%)	34.7	79.0	62.3	<0.001
Education (schooling/no schooling) (%)	41.3	55.6	50.3	0.03
Physical functional disability (mean ± SD)	5.37 ± 3.55	4.48 ± 2.98	4.82 ± 3.20	0.05
Exercise (%)	84.2	92.7	89.5	0.04
Hypertension (BP ≥ 140/90 or self-reported or on hypertensive medicine)	81.6	84.7	83.5	0.35
Hypertension (Self-reported)	64.5	64.5	64.5	0.55

**Table 2:** Distribution of blood pressure in the oldest old population.

Blood Pressure	Men		Women		Total		p
SBP (mean ± SD)	141.00 ± 23.01		141.35 ± 24.60		141.22 ± 23.95		0.91
DBP (mean ± SD)	81.20 ± 12.99		81.86 ± 11.67		81.61 ± 12.16		0.71
High SBP (%)	50.0		49.2		49.5		0.51
High DBP (%)	18.4		28.2		24.5		0.08
**JNC – 7**							
	**SBP**	**DBP**	**SBP**	**DBP**	**SBP**	**DBP**	---
Normal (%)	21.1	48.7	16.1	42.7	18.0	45.0	
Prehypertension (%)	28.9	31.6	33.9	29.0	32.0	30.0	
Stage 1 (%)	27.6	7.9	29.8	21.0	29.0	16.0	
Stage 2 (%)	22.4	11.8	20.2	7.3	21.0	9.0	
**Diagnosed and undiagnosed hypertension in the study population**							
Diagnosed HT (%)	79.0		81.9		80.0		0.39
Undiagnosed HT (%)	21.0		18.1		19.2		0.39
**Controlled and uncontrolled hypertension in the individuals who are taking medicines for hypertension (n=104)**							
Controlled with treatment (%)	44.4		47.1		46.2		0.43
Uncontrolled with treatment (%)	55.6		52.9		53.8		0.48
**Controlled and uncontrolled hypertension in the hypertensive individuals who are NOT taking medicines for hypertension (n =63)**							
Controlled without treatment (%)	76.9		73.0		74.6		
Uncontrolled without treatment (%)	23.1		27.0		25.4		0.40

**Table 3:** Correlates of hypertension in men and women aged ≥ 80 years.

Correlates	Men (N=76)			Women (N=124)			Total (N=200)			P-value (between men & women with HT)
	HT (N = 62)	No HT (N = 14)	P value	HT (N = 105)	No HT (N=19)	P-value	HT (N=167)	No HT (N=33)	P-value	
**Demographic factors and anthropometric measurements**										
Age (mean ± SD)	83.52 ± 4.10	84.93 ± 3.68	0.21	83.23 ± 3.88	83.28 ± 3.08	0.95	83.34 ± 3.95	84.00 ± 3.40	0.33	0.65
Sex (%)	37.1	42.4	-	62.9	57.6	-	83.5	16.5	0.35	
Living single (%)	31.1	50.0	0.15	80.0	73.7	0.36	62.0	63.6	0.51	<0.001
No education (%)	42.6	35.7	0.43	52.4	73.7	0.06	48.8	57.6	0.23	0.14
Smoking (%)	59.7	71.4	0.30	15.7	31.6	0.09	32.3	48.5	0.05	<0.001
Alcohol (%)	59.7	50.0	0.35	11.4	31.6	0.03	29.3	39.4	0.17	<0.001
BMI (mean ± SD)	22.86 ± 4.40	19.84 ± 2.92	0.006	23.56 ± 4.72	21.13 ± 4.56	0.06	23.31 ± 4.61	20.69 ± 3.97	0.002	0.35
Height (mean ± SD)	162.70 ± 9.02	161.06 ± 6.70	0.46	150.24 ± 6.59	145.93 ± 8.22	0.01	154.75 ± 9.63	152.27 ± 10.67	0.23	<0.001
Weight (mean ± SD)	61.11 ± 15.13	47.98 ± 17.20	0.007	58.02 ± 43.46	45.77 ± 12.22	0.02	59.14 ± 35.84	46.70 ± 14.29	0.001	0.51
Waist circumference (mean ± SD)	89.49 ± 18.35	84.36 ± 9.88	0.17	86.54 ± 12.99	80.11 ± 12.06	0.05	87.61 ± 15.16	81.89 ± 11.23	0.01	0.28
Hip circumference (mean ± SD)	94.97 ± 11.48	88.69 ± 10.61	0.07	98.81 ± 11.13	12.75 ± 13.60	0.04	97.42 ± 11.38	91.04 ± 12.41	0.01	0.03
Waist hip ratio (mean ± SD)	0.93 ± 0.13	0.95 ± 0.10	0.62	0.87 ± 0.09	0.86 ± 0.05	0.50	0.89	0.90	0.84	0.001
Systolic BP (mean ± SD)	145.31 ± 22.91	121.96 ± 10.66	<0.001	145.51 ± 23.98	118.68 ± 11.73	<0.001	145.3 ± 23.52	120.8 ± 11.24	<0.001	0.95
Diastolic BP (mean ± SD)	83.28 ± 12.93	72.00 ± 8.82	0.006	83.07 ± 11.84	75.21 ± 8.14	<0.001	83.15 ± 12.21	73.85 ± 8.45	<0.001	0.91
**Comorbidities**										
Haemoglobin (mean ± SD)	12.02 ± 1.87	12.32 ± 1.51	0.80	11.19 ± 1.58	10.24 ± 1.45	0.01	11.56 ± 1.76	11.12 ± 1.79	0.45	0.001
Diabetes self-reported ± high blood sugar (%)	25.8	0.00	0.02	23.3	11.1	0.23	24.20	6.2	0.01	0.42
Depression (mean ± SD) (GDS ≥ 5)	6.32 ± 5.02	7.00 ± 3.88	0.58	7.94 ± 5.01	9.57 ± 4.59	0.17	7.34 ± 5.06	8.48 ± 4.43	0.19	0.04
Depression (%)	38.7	57.1	0.16	55.2	73.7	0.09	49.1	66.7	0.02	0.02
Frailty (%)	77.4	92.9	0.17	83.8	89.5	0.41	81.4	90.9	0.14	0.20
CVD (%)	88.7	92.9	0.54	91.4	78.9	0.11	90.4	84.8	0.25	0.37
Anaemia (%)	62.9	50.0	0.27	61.9	100	<0.001	62.3	78.8	0.05	0.51
Arthritis (%)	38.7	35.7	0.54	49.5	26.5	0.04	45.5	30.3	0.07	0.11
Cognitive (mean ± SD)	19.95 ± 9.39	19.14 ± 7.90	0.74	18.14 ± 7.54	15.26 ± 8.65	0.18	18.81 ± 8.29	16.90 ± 8.44	0.24	0.17
Cognitive impairment (%)	53.2	64.3	0.32	70.5	84.2	0.17	64.1	75.8	0.13	0.01
Overweight/Obesity (%)	50.0	15.4	0.02	45.1	27.8	0.13	46.9	22.6	0.009	0.33
**Functional disabilities**										
Grip Strength (mean ± SD)	9.06 ± 9.80	6.60 ± 7.38	0.30	2.84 ± 4.46	3.10 ± 4.64	0.82	5.15 ± 7.54	4.59 ± 6.11	0.64	0.001
Short Physical performance Battery SPPB (%) ≤ 9	85.5	92.9	0.41	100	100	0.28	99.0	94.0	0.62	0.001
Gait speed (mean ± SD) (time seconds)	9.38 ± 4.82	7.88 ± 2.23	0.09	10.54 ± 4.95	10.87 ± 6.67	0.85	10.12 ± 4.92	9.53 ± 5.32	0.58	0.16
Low energy (%)	51.6	78.6	0.04	65.7	78.9	0.19	60.5	78.8	0.02	0.05
Asthma (%)	27.4	28.6	0.58	24.8	5.3	0.04	25.7	15.2	0.13	0.41
Stroke (%)	41.9	42.9	0.58	70.5	47.4	0.04	59.9	45.5	0.04	<0.001
Poor balance (%)	53.2	35.7	0.18	65.7	63.2	0.51	61.1	51.5	0.09	0.07
ADL (%)	72.6	85.7	0.25	92.4	84.2	0.22	85.0	84.8	0.57	0.001
Distance walked per day (KM) (mean ± SD)	1.38 ± 1.19	1.19 ± 0.96	0.54	0.91 ± 0.67	0.71 ± 0.48	0.11	1.08 ± 0.92	0.96 ± 0.74	0.94	0.002
Sleep quality (PQSI) (mean ± SD)	6.82 ± 3.50	7.57 ± 2.82	0.40	8.05 ± 3.73	7.26 ± 3.54	0.38	7.59 ± 3.69	7.39 ± 3.21	0.75	0.03
Poor sleep quality (%)	67.7	85.7	0.04	80.0	68.4	0.20	75.4	75.8	0.58	0.05
Heart rate (mean ± SD)	77.28 ± 12.67	82.86 ± 10.56	0.09	77.61 ± 12.33	87.26 ± 11.91	0.002	77.55 ± 12.53	85.58 ± 11.47	0.001	0.87

χ^2^ statistics, *t-test statistics

**Table 4:** Logistic regression predicting odds of hypertension in the total population.

Risk factors	Total population	
	OR	95% CI
Body Mass Index ( per 1SD increase)***	1.92	1.17–3.16
Heart Rate (per 1SD increase)***	0.44	0.27–0.71
Diabetes**	6.02	1.24–29.11
Asthma**	3.59	1.05–12.29

Backward stepwise logistic regression:

** p<0.05;

*** p<0.01

**Table 5:** Logistic regression predicting odds of hypertension in men.

Risk factors	Men	
	OR	95% CI
Body Mass Index (per 1SD increase) **	2.54	1.06 – 6.07
Low Energy	0.30	0.72 – 1.31

Backward stepwise logistic regression:

** p<0.05

**Table 6:** Logistic regression predicting odds of hypertension in women.

Risk factors	Women	
	OR	95% CI
Haemoglobin (per 1SD increase)**	2.02	1.01 – 4.08
Height (per 1SD decrease)**	2.52	1.05 – 6.09
Arthritis**	4.48	1.19 – 16.83
Asthma	5.57	0.64 – 48.13
Heart Rate (per 1SD increase)**	0.47	0.24 – 0.92

Backward stepwise logistic regression:

** p<0.05
